# Nuclear DNA Amount in a Diverse Collection of *Linum usitatissimum* Accessions

**DOI:** 10.3390/ijms27198613

**Published:** 2026-09-26

**Authors:** Gülru Yücel, Ömer Faruk Çatal, Şahane Funda Arslanoğlu

**Affiliations:** 1 Department of Agricultural Biotechnology, Faculty of Agriculture, Ondokuz Mayis University, 55139 Samsun, Türkiye; 2 Department of Agricultural Biotechnology, Graduate School of Education, Ondokuz Mayıs University, 55200 Samsun, Türkiye; omerfcatal@gmail.com; 3 Department of Field Crops, Faculty of Agriculture, Ondokuz Mayis University, 55200 Samsun, Türkiye; farslanoglu@omu.edu.tr; 4 Department of Agriculture and Breeding, Hemp Research Institute, Ondokuz Mayis University, 55200 Samsun, Türkiye

**Keywords:** *L. usitatissimum* collection, flow cytometry, nuclear DNA content, chromosome

## Abstract

Flax (*Linum usitatissimum*) is an important member of the genus *Linum*. *L. usitatissimum* has been commonly cultivated as a fiber or oil source. Despite its economic significance, comprehensive genome analyses encompassing a wide range of genotypes remain limited. Nuclear DNA content is defined as the amount of DNA within the nuclei of a eukaryotic organism. The elucidation of the nuclear DNA content may provide valuable insights for genetic studies, biodiversity studies, conservation efforts, and breeding programs, thereby reinforcing the significance of its estimation. Nuclear DNA content variation may arise under different environmental conditions, and its potential adaptive role has attracted considerable interest. In the present study, isolated intact nuclei were stained with propidium iodide as a DNA stain and flow cytometry analyses were performed to estimate the nuclear DNA content of two varieties and 40 accessions from geographically distant locations. FCM analyses revealed that the nuclear DNA content varied from 1.19 pg/2C to 1.37 pg/2C. This corresponds to a 15.13% difference in nuclear DNA content among the genotypes, with PI 182226 having the smallest genome and PI 194998 the largest genome. A minor variation was detected among the genotypes; however, accession PI 194998 showed statistically significant differences compared with some of the analyzed genotypes. The small intraspecific variation observed among the analyzed genotypes may indicate detectable genomic diversity, which could potentially be attributed to differences in the abundance of repetitive elements among these genotypes. The chromosome number of the selected genotypes was determined to rule out ploidy-level variation as a potential explanation of differences in nuclear DNA content, and all shared the expected same ploidy level. Geographical location (longitude and latitude) showed no statistically significant correlation with nuclear DNA content. The potential causes of the intraspecific variation are discussed in the text. The results obtained in this study may constitute a valuable genetic resource for subsequent molecular and breeding research aimed at improving this economically important crop.

## 1. Introduction

Flax (*Linum usitatissimum* L.) is an annual plant that belongs to the genus *Linum* and is an important member of the family Linaceae [1]. Flax is a self-pollinated plant [1] with a very low rate (0–5%) of outcrossing [2]. For over 10,000 years, flax has been cultivated in the Near East as an ancient crop [3,4]. Historically, two main types of cultivars have been developed, fiber flax and oilseed flax (linseed), corresponding to distinct end uses. More recently, dual-purpose cultivars suitable for both fiber and oil production have also been released [5]. Flax fibers are widely used in the textile and paper industries [6] and flax remains one of the most important natural fibers globally after cotton [7]. Linseed is known for its high ALA and also dietary fiber. Owing to linseed oil’s drying properties, it is used for several purposes such as in varnishes and flooring [5,8]. The genetic inheritance pattern for *L. usitatissimum* has been proposed to be a diploid [9] or tetraploid genome [10]. Previous experiments have reported that the chromosome number of *L. usitatissimum* is 2*n* = 30 [10,11]. Two different hypotheses have been proposed regarding the genome of *L. usitatissmum*. The tetraploid cultivated *L. usitatissimum* (2*n* = 30) and wild progenitor of its (*L. angustifolium*, 2*n* = 30) are hypothesized to have originated either through the hybridization of two diploid species with 2*n* = 16 chromosome number (a relative of modern *L. decumbens* and *L. grandiflorum*) followed by a process of diploidization, or hybridization between two diploid species, one of them representing the ancestor of modern *L. narbonense* with 2*n* = 14 chromosomes and the other diploid species with 2*n* = 14 chromosomes, which make its analysis of scientific interest [10]. Resolving these alternative scenarios is of significant interest for understanding flax evolution [9,10]. Flax has attracted increasing research interest and is considered a crop that remains relatively under-investigated [12]. Knowledge of related genetic diversity is a crucial step in designing suitable breeding strategies [13,14]. Therefore, understanding genetic diversity for sustainable flaxseed production is crucial to overcome climate fluctuation challenges and identify high-yielding genotypes [13,14]. A range of analyses of molecular markers, e.g., EST-SSR [14], IRAP [15], ISSR [16], RAPD [17], and SSR [18], has been conducted, including *L. usitatissimum*. Despite the substantial degree of variation reflected, it has been mentioned that further robust validation combining additional markers is required [14], which may highlight a need for further comprehensive studies. Recent comparative genomic analysis and pangenomic analyses have contributed to our understanding of the flax genome, and have facilitated the development of genomic and genetic tools that are useful for breeding and genetic studies, e.g., the identification of genetic markers for genotyping characterization and the development of genotypes adapted to climatic contexts [19]. The characterization of genotypes [19] and diversity analyses of germplasms provide breeders with improved options [20], such as identifying divergent genotypes that could represent a promising approach to creating genetic variability in *L. usitatissimum* for future breeding programs [21]. Thus, the reported differences in nuclear DNA analyses are quite noteworthy in *L. usitatissimum,* as they indicate substantial variations ranging from 0.764 pg/2C (picogram) [9] to 1.53 pg/2C [22,23]. Furthermore, environmentally induced changes, including the development of large and small genotrophs accompanied by variations in nuclear DNA content in some individuals, have been documented [22,23,24]. Given that nuclear DNA content has been estimated using different experimental approaches, standards, methods, conditions, genotypes, etc., in previous research, it is important to understand nuclear content and its variation [25]. Nuclear DNA content represents a key genomic attribute in various research fields, e.g., systematics and conservation, and in biodiversity studies [26,27]. The 2C value represents the amount of DNA found in a diploid somatic nucleus [28,29]. Flow cytometry is the most commonly used method to estimate nuclear DNA content in plant genomes due to its ease, accuracy, cost-effectiveness, and stability when used to undertake subsequent large-scale analyses [29,30]. An increasing number of studies have reported intraspecific variations in a wide range of other species, including *Chenopodium quinoa* [31], *Brachypodium hybridum* [32], and *Rorippa indica* [33].

On the other hand, the change in plant genome size is associated with several environmental and geographical factors [34]. For instance, longitude and latitude have different climatic variables, e.g., rainfall, precipitation, and temperature, that may exert different influences on plant traits and evolution [35]. Intraspecific variations in genome size have been proposed to be associated with the adaptive strategy under stressful conditions [36,37,38]. Moreover, intraspecific variation may serve as a detectable category of genomic diversity that could be valuable for use in breeding and molecular studies [24]. Like in any other crop, flax faces challenges with productivity, tolerance to abiotic stress, disease tolerance, and adaptation to climate change [19], particularly drought [13]. Overcoming the challenges mentioned relies on maintaining genetic diversity in both natural environments and gene banks [39,40]. Therefore, the detection of the nuclear DNA content polymorphism may constitute a significant parameter for the characterization of genetic resources [24,40,41]. 

This research aimed to estimate the nuclear DNA content among 40 accessions and two varieties of *L. usitatissimum* from different localities in order to test whether *L. usitatissimum* exhibits intraspecific polymorphisms in nuclear DNA content. Furthermore, in order to rule out chromosomal variation as a possible underlying source of the variation in nuclear DNA content, the chromosome numbers of some individuals were determined.

## 2. Results

In the present study, nuclear DNA content was analyzed in 42 genotypes (40 accessions and 2 varieties). All accessions were analyzed with three repetitions to estimate the mean nuclear DNA content by flow cytometry. The replicated measurements within each analyzed genotype were consistent. The standard deviation of nuclear DNA content varied from 0.01 to 0.08 among the genotypes. Nuclear DNA content varied from 1.19 pg/2C to 1.37 pg/2C. These data correspond to a 15.13% difference in nuclear DNA content among the genotypes, with PI 182226 from Syria having the smallest genome (1.19 pg/2C) and PI 194998 from Japan having the largest genome (1.37 pg/2C) (Table 1).

Clearly defined histograms were obtained following the flow cytometry analyses. *V. sativa* L. is a suitable reference plant since its G1 peak is clearly distinguished from the G1 peak of *L. usitatissimum* (Figure 1). Most of the samples have CV < 5% or around 5%. Approximately 2000 nuclei were analyzed to produce the presented histogram (Figure 1). 

Since the normality assumption was not met in some groups (*p* < 0.05), the non-parametric Kruskal–Wallis test was performed, which has non-parametric assumptions. The non-parametric Kruskal–Wallis rank-sum test indicated highly significant disparities in median DNA content throughout the accessions (*p* < 0.001). To clarify these differences and establish boundaries, we conducted Dunn’s post-hoc test with a Benjamini–Hochberg false discovery rate (FDR) correction. The multiple comparisons helped categorize the genotypes into five overlapping significance groups (a, ab, abc, bc, and c), indicating a predominantly continuous distribution of DNA content throughout the majority of the population. PI 194998 demonstrated the highest DNA content, distinctly categorizing it into a unique group (“a”).

In contrast, accession PI 182226 exhibited the lowest relative DNA content, positioning it at the lower end of the spectrum (group “c”). The remaining 40 accessions constituted a wide continuum of intermediate values lacking distinct statistical boundaries. A boxplot illustrates the distribution of nuclear DNA content across the 42 accessions of the analyzed *L. usitatissimum* genotypes (Figure 2).

To further investigate the similarity of structure among accessions, hierarchical cluster analysis was conducted using Euclidean distances based on population means and the UPGMA algorithm (Figure 3). The highest nuclear DNA content was measured for the PI 194998 genotype (≈1.36–1.38), which was statistically in the top group (“group a”), whereas the lowest DNA content was measured for the PI 182226 genotype (≈1.16–1.22), which was in group “c” based on post-hoc analyses. The uniqueness of the upper-bound outlier (PI 194998) was firmly validated by an unsupervised clustering analysis solely reliant on DNA content. The algorithm identified an optimal cluster count of k = 2, corroborated by a substantial cophenetic correlation coefficient of 0.839, signifying a robust alignment between the dendrogram and the foundational distance matrix. This analysis distinctly divided the population into two markedly unequal groups: Cluster 1 included 41 genotypes, reflecting the core population consensus, whereas Cluster 2 comprised solely the single, high-DNA accession PI 194998 (Figure 3).

To test if the observed variation in DNA content is correlated with the geographical locations of the accessions, we performed a Spearman’s rank correlation analysis using the geographic coordinates (latitude and longitude) of countries. No significant correlation was observed between DNA content and either the latitude (*p* = 0.3826, *n* = 40) or the longitude (*p* = 0.1429, *n* = 40) (Figure 4 and Figure 5).

The mitotic chromosome numbers of *L. usitatissimum* samples were correlated with their obtained nuclear DNA content by counting the mitotic chromosomes of six different genotypes with a microscope. Cytological experiments revealed that the chromosome numbers of the examined individuals (Yılmaz, Sarı 85, PI 91037, PI 176623, PI 194998 and PI 182226) were 2*n* = 30 (Figure 1). All analyzed genotypes of *L. usitatissimum* were assumed to have the same ploidy level, since they have shown similar 2C nuclear DNA contents.

## 3. Discussion

Genetic, physical, and cytological maps, along with their incorporation, are important characteristics for investigating the genetic features of this significant plant and grounding further progress in flax breeding [42]. It is crucial to elucidate the nuclear genome size, as it provides valuable genome information related to ploidy level [40], intraspecific variation [25,31,32], genetic stability [43], the reproduction pathway, etc. [44]. Nuclear DNA content analyses have been performed in various plant species using flow cytometry for different purposes, e.g., ploidy-level detection in *Urochloa* s. l. [45], intraspecific variation in *Artemisia argyi* [41], and the type of reproduction *in Commiphora wightii* [46]. In this study, flow cytometry analyses were performed with a large number of *L. usitatissimum* accessions to detect 2C nuclear DNA content and to assess potential variation among the analyzed accessions using propidium iodide staining by flow cytometry. Limited research has been performed to detect nuclear DNA content in *L. usitatissimum,* e.g., 1.32 pg/2C and 1.53 pg/2C [22], and from 1.32 to 1.53 pg [23] and 0.764 pg/2C [9]. It has been reported that specific environmental conditions, such as subjection to fertilizer and temperature, may be associated with the divergence of DNA content in some *L. usitatissimum* individuals, including some genotrophs as well, indicating differences in DNA amount of up to 16% [22,23,47,48]. Polymorphisms of the insertional LIS−1 sequence [49] and altered copy numbers of 5S DNA [50] or other repetitive elements may be responsible for the nuclear DNA content variation [51]. In the presented study, the nuclear DNA contents of 42 genotypes were found to vary from 1.19 pg/2C to 1.37 pg/2C. In contrast, some previously obtained values range slightly higher and lower, varying from 0.764 pg/2C [9] to 1.53 pg/2C [23]. Timmis and Ingle (1973) found nuclear DNA contents of 1.32 pg/2C and 1.53 pg/2C when using the Feulgen densitometry method with *Allium cepa* as an internal standard plant under specific environmental conditions [22], whereas Wang et al. (2012) reported a lower value of 0.764 pg/2C, using *Raphanus sativus* as a reference plant, by flow cytometry [9]. The differences between those reported data and our results may be attributed to differences in the applied staining protocol [52], different internal standards used [53], repetitive elements [54], the analyzed genotype [25,29], and intraspecific variations [55]. Furthermore, minor and non-statistically significant variations in nuclear DNA content among the eight flax varieties under field conditions have previously been reported, which are consistent with the findings of the present study [48]. Several studies have reported associations between genome size variation and ecological conditions, including specific geographic factors, e.g., latitude. However, a straightforward relationship between environment and intraspecific variation, such as altitude and latitude, is still not clear [56]. Furthermore, environmental variables such as temperature and precipitation may be associated with variations in genome size [37]. The presented study was conducted based solely on latitude and longitude at the country level. However, comprehensive analyses on factors such as detailed location and environmental conditions are suggested so as to elucidate the potential influence of environmental conditions [22,37,38,56]. In our findings, neither latitude nor longitude showed a statistically significant correlation with nuclear DNA content. The obtained findings agree with those in the literature; for instance, longitude and latitude did not show any statistically significant correlation with nuclear DNA content in *Phaseolus vulgaris* [55] and *Prunus armeniaca* wild accessions [57]. Besides this, the nuclear DNA content variation was not correlated with the collection site of *C. quionia* [31]. However, several studies reporting findings that differ from our results have also been documented in the literature [58,59]. On the other hand, species with larger or smaller genomes may display an advantage in different environments [33,60], with enhanced reproductive activity [33] or competitive advantages [60]. However, the adaptive role of genome size variation under stressful environments remains a subject of ongoing debate [61]. These hypotheses underscore the importance of genome size [33]. In the current study, cytological analyses confirmed that the examined accessions share the same ploidy level (Figure 5). Based on their comparable DNA contents, the remaining samples were assumed to have the same ploidy level. The obtained results of the cytological investigations in the presented study are in agreement with the known chromosome number of *L. usitatissimum* [42,62]. Since all analyzed genotypes were assumed to have the same ploidy level, the observed differences (15.13%) in nuclear DNA content may be associated with the variation in repetitive elements [31,33]. This finding is in agreement with the results of several previous studies, which reported variations in nuclear DNA content in species such as *C. quinoa* (5.9%) [31] and hexaploid *Rorippa* (15.8%) [33]. Variations in nuclear DNA content might arise from the amplification or elimination of repetitive elements, such as the TE bursts mentioned as the potential source of genome expansion [33] and variations related to repetitive DNA sequences, particularly Ty1-*copia* retrotransposons (Bianca) and 45S [33], small deletions [63] and illegitimate recombination could be the underlying reason for genomic DNA loss [64]. In *L. usitatissimum*, the diversity and abundance of *Copia* LTR elements have been proposed to contribute to shaping its genome [65]. Moreover, genome size variation within the genus *Linum* has been reported to be primarily driven by transposable elements, particularly a recent burst of long terminal retrotransposons (LTR) [66]. Collectively, our findings suggest that the observed variation in nuclear DNA content among *L. usitatissimum* genotypes may, at least in part, be associated with the contribution of repetitive sequences [33,63,64,65,66]. The variation among some of the analyzed genotypes, reaching 15.13%, may suggest that part of the obtained differences could be attributed to genomic diversity [24]. Various molecular marker-based analyses have provided genetic diversity among *L. usitatissimum* accessions, such as ISSR [16] and RAPD [17]. In this regard, polymorphism in nuclear genome size and data obtained from the molecular marker analyses may provide complementary evidence of the genetic diversity, which could be useful for future studies [24]. 

## 4. Materials and Methods

### 4.1. Experimental Materials 

A total of 42 genotypes (40 accessions and 2 cultivars, Sarı85 linseed, Yılmaz fiber seed of *L. usitatissimum*) were analyzed (Table 2). USDA (The United States Department of Agriculture) accession numbers and information about the analyzed samples are listed in Table 2. Materials were grown from seeds in a greenhouse facility of the Faculty of Agriculture Ondokuz Mayıs University. All plants were grown in greenhouse conditions at about 24 °C (room temperature), 80% humidity, and 12 h of light per day. During this period, daily irrigation and maintenance were performed.

### 4.2. Preparation of the Samples for Nuclear DNA Content Analyses

The 2C nuclear DNA contents of the varieties and accessions were measured using flow cytometry (Partec CyFlow^®^ Space, Munster, Germany) in the Plant Genetics and Cytogenetics Laboratory at Tekirdağ Namik Kemal University. Thirteen accession’s nuclear DNA content obtained from Çatal (2026) [67]. As an internal standard, *Vicia sativa* (3.65 pg/2C DNA; 40) was used in the analyses. Fresh, healthy, and fully developed upper young leaves were collected from plants with at least 8–10 leaves and used for flow cytometry analyses. The intact nuclei suspensions derived from the samples were prepared using the CyStain^®^ PI Precise *p* commercial kit, which was manufactured by Sysmex Partec GmbH (Münster, Germany). Following the procedure, the leaves of the internal standard and sample materials were chopped with a razor blade in Petri dishes containing 500 µL of nuclei extraction buffer. The sample solution was transferred into a tube using a filter (30 μm CellTrics). Following the filtration, the prepared 2 mL staining buffer containing propidium iodide was added to the tube. Flow cytometry analyses were performed using three different individuals for each genotype. The prepared samples were incubated for approximately 1 h at room temperature in dark conditions. Samples were analyzed by flow cytometry (Partec CyFlow Space flow cytometer, Munster, Germany). The nuclear DNA contents of the accessions were calculated based on the ratio of the G1 peak means of the internal standard (*V. sativa*) to those of each *L. usitatissimum* sample using the following formula [68].

Sample nuclear DNA content = Fluorescence intensity of the sample (mean of the G1 peak) Fluorescence intensity of the standard (mean of the G1 peak) × DNA content of the standard

### 4.3. Statistical Analyses

The statistical analyses of the samples were performed using Python (version 3.12.3). Statistical tests were conducted with statsmodels (version 0.14.4), scipy (version 1.13.1), and libraries [69,70]. The data manipulation and visualization were conducted using matplotlib (version 3.9.1), pandas (version 2.1.4), and seaborn [71,72]. For each genotype, three different individuals were analyzed to estimate nuclear DNA content. The mean DNA was calculated and used for the statistical analyses. The Shapiro–Wilk test was used to assess normality [73]. Because the nuclear DNA content among genotypes did not follow a normal distribution, the non-parametric Kruskal–Wallis test was applied to evaluate the differences among the genotypes at a significance level of 5% [74,75]. Subsequently, Dunn’s post-hoc test with Benjamini–Hochberg false discovery rate (FDR) correction was performed to identify specific pairwise differences and assign statistical groupings [76,77]. To assess relationships among the 42 genotypes, a hierarchical dendrogram analysis Unweighted Pair Group Method with Arithmetic Mean (UPGMA) was conducted [78,79]. Spearman’s rank correlation was used to test whether nuclear DNA content variation was correlated with the latitude and longitude of the countries. Two accessions (PI91037** and PI91031**) were discarded from Spearman’s rank correlation analyses due to the limited information available.

### 4.4. Somatic Chromosome Slide Preparations

About 2 cm-long young and healthy roots were collected from six genotypes of *L. usitatissimum*—randomly selected genotypes with different nuclear DNA contents (Yılmaz, Sarı 85, PI 91037, PI 176623, PI 182226 and PI 194998)— to prepare somatic chromosome slides. Chromosome number of Sarı 85 cultivar was obtained from Çatal (2026; [67]). The collected roots were pretreated in ice-cold water for 24 h and fixed in ethanol/glacial acetic acid (3:1). The fixed root samples were stored at 4 °C till use. The preparations of somatic chromosome slides were performed as explained by Hasterok et al. (2004) [80]. Briefly, prepared roots were washed for 20 min in citrate buffer (0.01 M) before digestion in a mixture of enzymes containing 20% (*v*/*v*) pectinase (Sigma, St. Louis, MO, USA) and 1% (*w*/*v*) cellulase ‘Onozuka R-10’ (Sigma, St. Louis, MO, USA) for 2 h at 37 °C in an incubator. The root meristems were dissected under a stereo microscope. The dissected root meristems of the analyzed samples were put onto the microscope slide in a drop of 45% acetic acid and squashed. The coverslips of the prepared slides were removed following freezing. Two replicates were performed for each genotype, and five metaphase plates from each were observed. The prepared slides were air-dried overnight and then stained with 2.5 ng/µL of DAPI (4,6-diamidino-2-phenylindole dihydrochloride; Sigma, St. Louis, MO, USA). Somatic chromosome slides were observed using a 100× objective under an Olympus BX51 light microscope (Olympus Corporation, Tokyo, Japan). The images of the somatic chromosomes were captured using a Spot RT Slider CCD digital camera (SPOT Imaging, Sterling Heights, MI, USA) attached to the microscope. The image processing of the prepared slides was performed using Wasabi (Hamamatsu Photonics K.K., Shizuoka, Japan).

## 5. Conclusions

To summarize, we conducted estimations of the nuclear DNA content, based on extensive sampling, of *L. usitatissimum* via flow cytometric analyses. No significant correlation was found between longitude and latitude and the nuclear DNA content of *L. usitatissimum* accessions. The accession PI 194998 showed a statistically significant difference from some other accessions included in the analyses. Intraspecific variation may exist and potentially be attributable to differences in repetitive elements that contribute to variations in nuclear DNA content, suggesting minor genetic variation in certain accessions. Thus, minor reported variations could be useful for use in future detailed molecular and cytogenetic analyses, and may improve the selection of genotypes for breeding programs focused on improving this valuable crop. Further comprehensive analyses, such as repetitive element sequencing and high-resolution karyotyping, could be valuable in offering deeper insights into the genome of *L. usitatissimum*. In addition, a large number of samples and more detailed environmental factors suggested to assess the potential relationship between nuclear DNA content and adaptation.

## Figures and Tables

**Figure 1:**
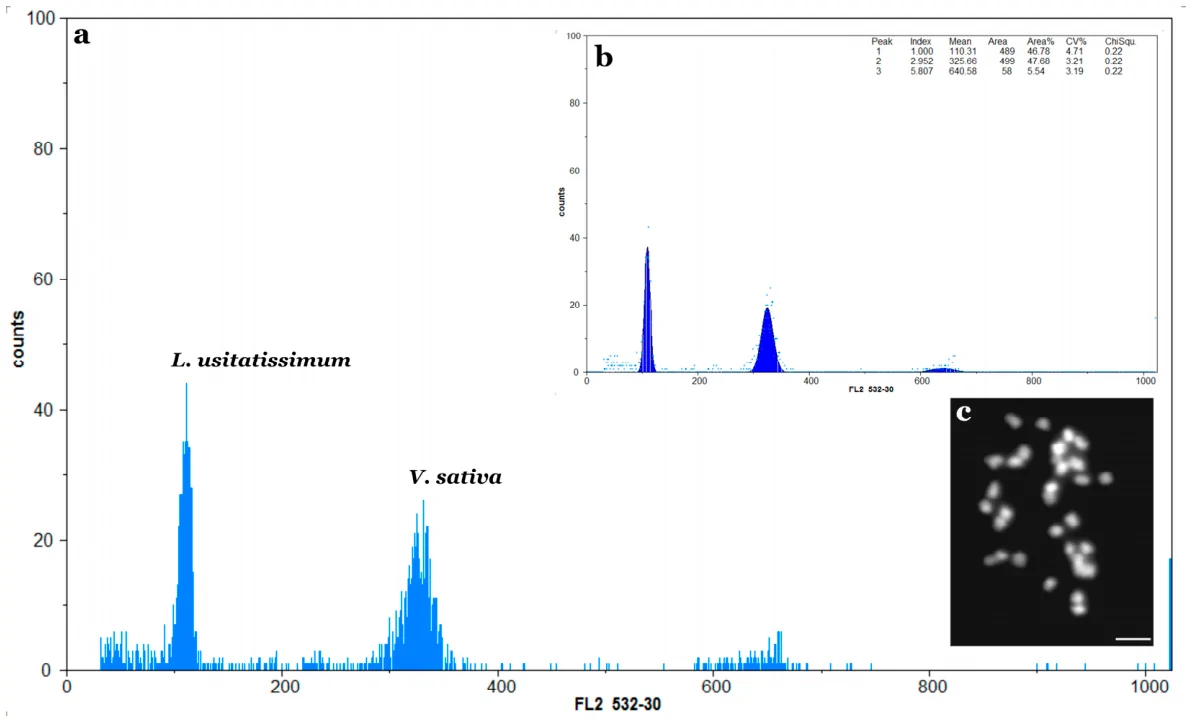
The flow cytometry histogram indicates the relative positions of the G1 peaks of *L. usitatissimum* (PI 175770) and the standard (**a**). The flow cytometry results were processed using the FloMax analysis software (Sysmex Partec GmbH, Münster, Germany), indicating mean fluorescence intensity data (**b**). Detected mitotic chromosomes of *L. usitatissimum* (2*n* = 30, PI 176623) (**c**). Bar 5 μm.

**Figure 2:**
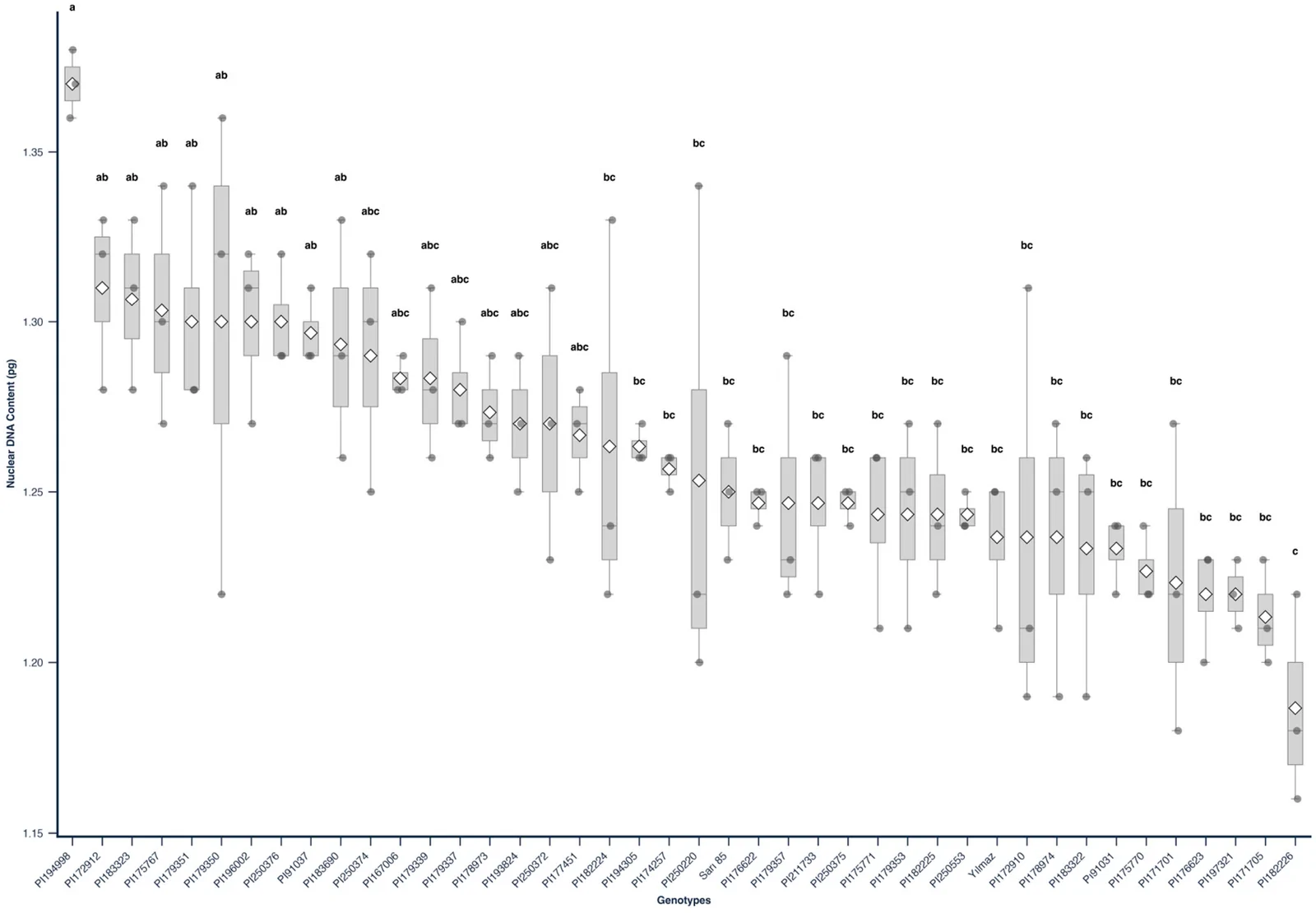
The box plot indicates the variation in nuclear DNA content. The dots show the individual measurements, the diamond (◊) indicates the mean, and the grey line in each box indicates the median. The letters (from a to c) above the boxes indicate statistically significant differences according to post hoc analysis; genotypes sharing the same letter do not differ significantly.

**Figure 3:**
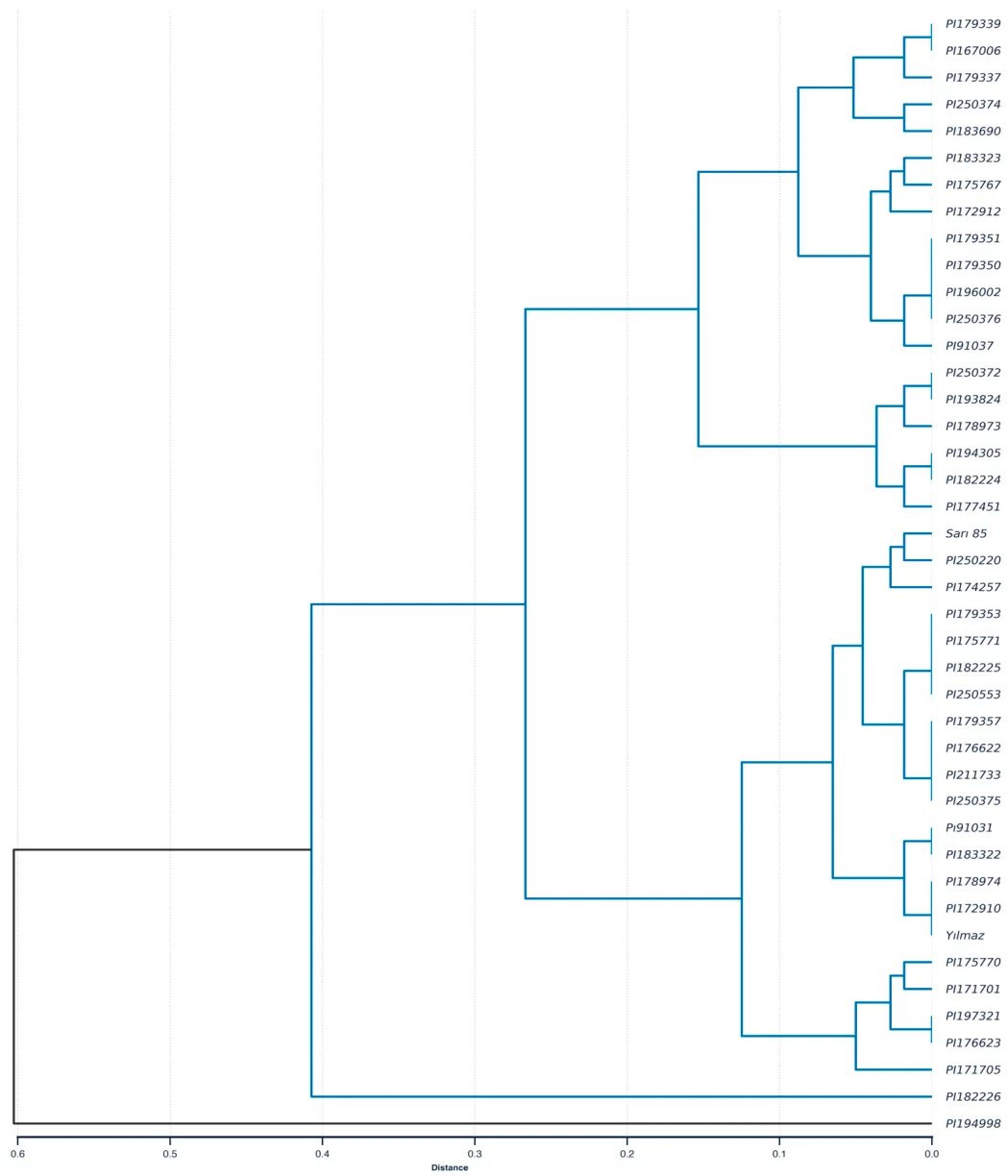
The dendrogram indicates the relations of genotypes based on nuclear DNA content analyses.

**Figure 4:**
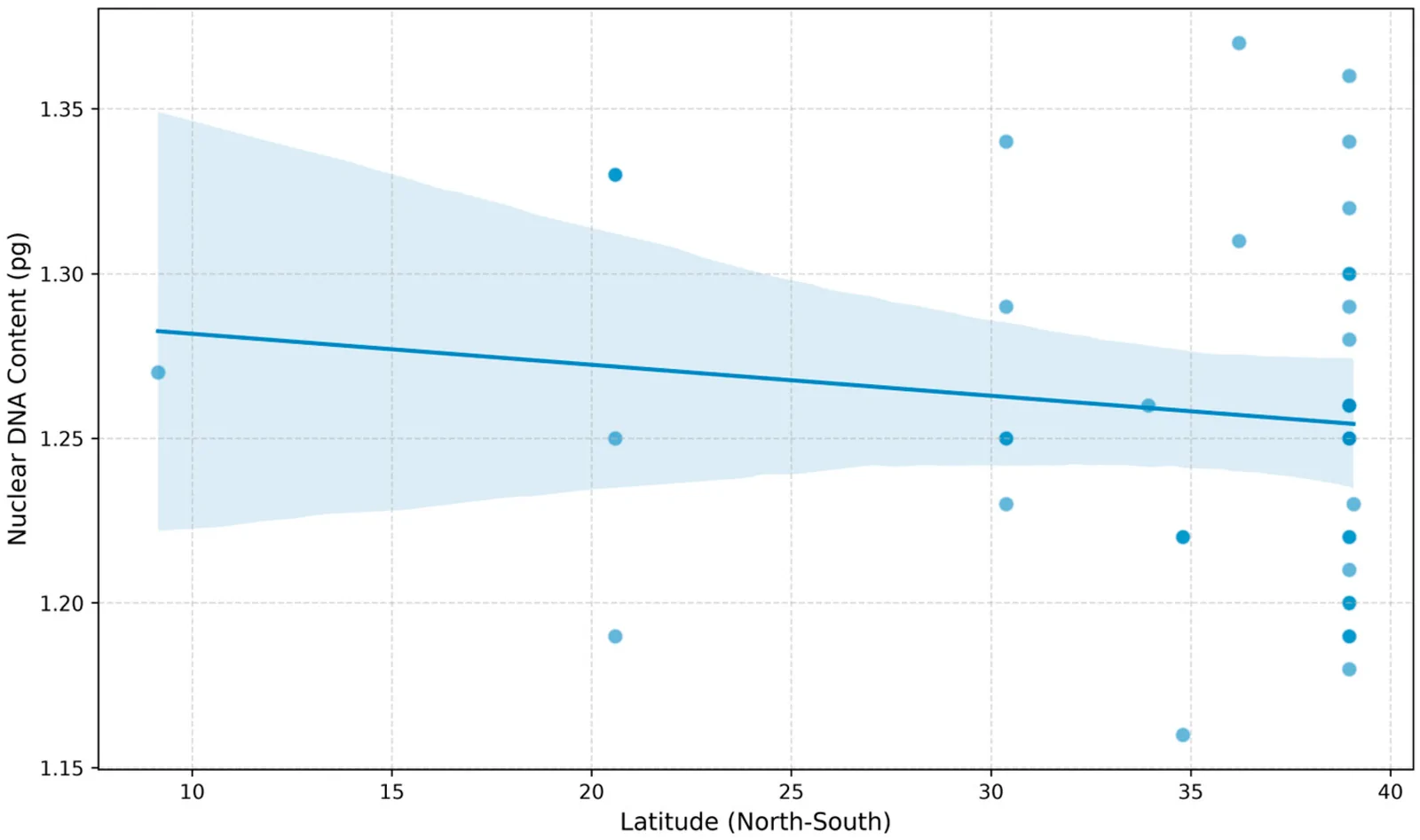
Results of the Spearman correlation analysis between latitude (North–South) and nuclear DNA content. The blue circles indicate (○) the accession numbers. The blue line indicates the correlation.

**Figure 5:**
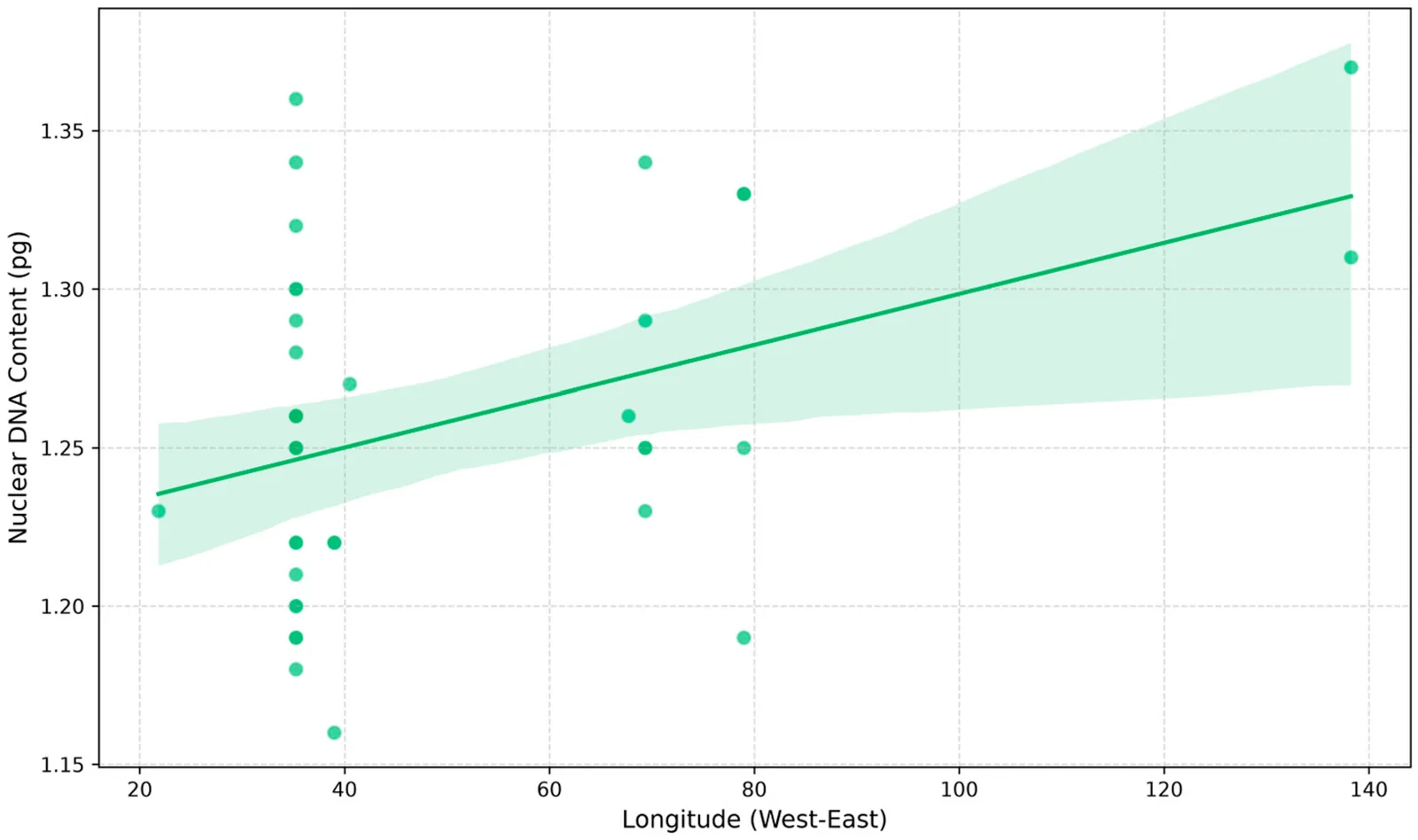
Results of the Spearman correlation analysis between the longitude (West–East) and nuclear DNA content. The green circles indicate (○) accession number. The green line indicates the correlation.

**Table 1:** Nuclear DNA contents of the analyzed samples.

Accesions	Mean DNA Content	SD	Letter Group	Accesions	Mean DNA Content	SD	Letter Group
PI 194998	1.37	0.01	a	PI 250220	1.25	0.08	bc
PI 172912	1.31	0.03	ab	Sarı85	1.25	0.02	bc
PI 183323	1.31	0.03	ab	PI 176622	1.25	0.01	bc
PI 179351	1.30	0.03	ab	PI 179357	1.25	0.04	bc
PI 175767	1.30	0.04	ab	PI 211733	1.25	0.02	bc
PI 179350	1.30	0.07	ab	PI 250375	1.25	0.01	bc
PI 196002	1.30	0.03	ab	PI 175771	1.24	0.03	bc
PI 250376	1.30	0.02	ab	PI 179353	1.24	0.03	bc
PI 91037	1.30	0.01	ab	PI 182225	1.24	0.03	bc
PI 183690	1.29	0.04	ab	PI 250553	1.24	0.01	bc
PI 250374	1.29	0.04	abc	Yılmaz	1.24	0.02	bc
PI 167006	1.28	0,01	abc	PI 172910	1.24	0.06	bc
PI 179339	1.28	0.03	abc	PI 178974	1.24	0.04	bc
PI 179337	1.28	0.02	abc	PI 183322	1.23	0.04	bc
PI 178973	1.27	0.02	abc	PI 91031	1.23	0.01	bc
PI 193824	1.27	0.02	abc	PI 175770	1.23	0.01	bc
PI 250372	1.27	0.04	abc	PI 171701	1.22	0.05	bc
PI 177451	1.27	0.02	abc	PI 176623	1.22	0.02	bc
PI 182224	1.26	0.06	bc	PI 197321	1.22	0.01	bc
PI 194305	1.26	0.01	bc	PI 171705	1.21	0.02	bc
PI 174257	1.26	0.01	bc	PI 182226	1.19	0.03	c

**Table 2:** Data on *L. usitatissimum* genotypes used for nuclear DNA content analysis.

Accessions	Top Name	Country	Accessions	Top Name	Country
PI 194998	CIli 1510	Japan	Sarı85 (linseed)		Türkiye
PI 172912	CIli 1369	Türkiye	PI 176622	CIli 1410	Türkiye
PI 183323	CIli 1466	India	PI 179357	CIli 1452	Türkiye
PI 179351	CIli 1446	Türkiye	PI 211733	CIli 1617	Afghanistan
PI 175767	CIli 1398	Türkiye	PI 250220	CIli 1932	Pakistan
PI 179350	CIli 1445	Türkiye	PI 250375	CIli 1943	Pakistan
PI 196002	CIli 1518	Japan	Yılmaz (fiber)		Türkiye
PI 250376	CIli 1944	Pakistan	PI 172910	CIli 1367	Türkiye
PI 183690	CIli 1468	India	PI 175771	CIli 1402	Türkiye
PI 250374	CIli 1942	Pakistan	PI 178974	CIli 1428	Türkiye
PI 167006	CIli 1337	Türkiye	PI 179353	CIli 1448	Türkiye
PI 179339	CIli 1434	Türkiye	PI 182225	CIli 1458	Syria
PI 179337	CIli 1432	Türkiye	PI 250553	CIli 1993	Pakistan
PI 177451	CIli 1423	Türkiye	PI 175770	CIli 1401	Türkiye
PI 178973	CIli 1427	Türkiye	PI 183322	CIli 1465	India
PI 193824	CIli 1497	India	PI 171701	CIli 1363	Türkiye
PI 250372	CIli 1940	Pakistan	PI 176623	CIli 1411	Türkiye
PI 174257	CIli 1391	Türkiye	PI 197321	CIli 1521	Greece
PI 182224	CIli 1457	Syria	PI 171705	CIli 1366	Türkiye
PI 194305	CIli 1502	Ethiopia	PI 182226	CIli 1459	Syria
PI 91037 **	CIli 637		PI 91031 **	CIli 641	

** Limited information available.

## Data Availability

The original contributions presented in this study are included in the article. Further inquiries can be directed to the corresponding author.

## References

[1] Zhao X., Yi L., Zuo Y., Gao F., Cheng Y., Zhang H., Zhou Y., Jia X., Su S., Zhang D. (2023). High-Quality Genome Assembly and Genome-Wide Association Study of Male Sterility Provide Resources for Flax Improvement. Plants.

[2] Dillman A.C. (1938). Natural crossing in flax. Agron. J..

[3] Zohary D., Hopf M., Weiss E. (2012). Domestication of Plants in the Old World—The Origin and Spread of Domesticated Plants in Southwest Asia, Europe, and the Mediterranean Basin.

[4] Fu Y.B. (2025). Flax domestication processes as inferred from genome-wide SNP data. Sci. Rep..

[5] Nag S., Mitra J., Karmakar P.G. (2005). An Overview on Flax (*Linum usitatissimum* L.) and its Genetic Diversity. Int. J. Agric. Environ. Biotechnol..

[6] Jhala A., Hall L.M. (2010). Flax (*Linum usitatissimum* L.): Current Uses and Future Applications. Aust. J. Basic Appl. Sci..

[7] Hazneci E., Arslanaoğlu Ş.F. (2021). Orta Karadeniz Bölgesinde Kırsal Alanlar İçin Keten Bir Şans Mı? Kârlılık Analizi ve Yapılabilirliği. J. Tekirdag Agric. Fac..

[8] Cloutier S. (2016). Reference Module in Food Science.

[9] Wang Z., Hobson N., Galindo L., Zhu S., Shi D., McDill J., Yang L., Hawkings S., Neutelings G., Datla R. (2012). The genome of flax (*Linum usitatissimum*) assembled de novo from short shotgun sequence reads. Plant J..

[10] Bolsheva N.L., Melnikova N.V., Kirov I.V., Speranskaya A.S., Krinitsina A.A., Dmitriev A.A., Belenikin M.S., Krasnov G.S., Lakunina V.A., Snezhkina A.V. (2017). Evolution of blue-flowered species of genus *Linum* based on high-throughput sequencing of ribosomal RNA genes. BMC Evol. Biol..

[11] Yadav H.K., Singh N., Singh B., Kaur V., Sawant S.V. (2025). Telomere-to-telomere genome assembly of linseed (*Linum usitatissimum* L.) for functional genomics and accelerated genetic improvement. Plant Biotechnol. J..

[12] Koçak M.Z., Zare S. (2026). Comparative Phytochemical and Fatty Acid Profiling of Flax (*Linum usitatissimum* L.) Cultivars Across Diverse Geographic Origins. Eur. J. Lipid Sci. Technol..

[13] Kaur V., Singh M., Wankhede D.P., Gupta K., Langyan S., Aravind J., Thangavel B., Yadav S.K., Kalia S., Singh K. (2023). Diversity of *Linum* genetic resources in global genebanks: From agro-morphological characterisation to novel genomic technologies—A review. Front. Nutr..

[14] Choudhary S.B., Sharma H.K., Kumar A.A., Chowdhury I., Maruthi R.T., Kak A. (2017). Genetic diversity spectrum and marker trait association for agronomic traits in global accessions of *Linum usitatissimum* L. Ind. Crops Prod..

[15] Smýkal P., Bačová-Kerteszová N., Kalendar R., Corander J., Schulman A.H., Pavelek M. (2011). Genetic diversity of cultivated flax (*Linum usitatissimum* L.) germplasm assessed by retrotransposon based markers. Theor. Appl. Genet..

[16] Uysal H., Fu Y.B., Kurt O., Peterson G.W., Diederichsen A., Kusters P. (2010). Genetic diversity of cultivated flax (*Linum usitatissimum* L.) and its wild progenitor pale flax (*Linum bienne* Mill.) as revealed by ISSR markers. Genet. Resour. Crop Evol..

[17] Fu Y. (2005). Geographic Patterns of RAPD Variation in Cultivated Flax. Crop Sci..

[18] Bhajantri V., Shakunthala N.M., Verulkar S.B., Saxena R.R., Vasudevan S.N., Macha S.I., Kisan B., Sahu H., Sonal U. (2020). Molecular Characterization and Diversity Analysis viz SSR Markers in Linseed (*Linum usitatissimum* L.) Germplasm. Chem. Eng..

[19] Demenou B.B., Ndar A., Pineau C.P., Hinsinger D.D., Marande W., Hourcade D., Faivre-Rampant P. (2025). Chromosome-scale assembly of European flax (*Linum usitatissimum* L.) genotypes and pangenomic analysis provide genomic tools to improve breeding. BMC Genom..

[20] Hoque A., Fiedler J.D., Rahman M. (2020). Genetic diversity analysis of a flax (*Linum usitatissimum* L.) global collection. BMC Genom..

[21] Singh N., Agarwal N., Yadav H.K. (2019). Genome-wide SNP-based diversity analysis and association mapping in linseed (*Linum usitatissimum* L.). Euphytica.

[22] Timmis J.N., Ingle J. (1973). Environmentally induced changes in rRNA gene redundancy. Nat. New Biol..

[23] Cullis C. (1976). Environmentally induced changes in ribosomal RNA cistron number in flax. Heredity.

[24] Durrant T., Timmis J.N. (1973). Genetic control of environmentally induced changes in Linum. Heredity.

[25] Yücel G., Arslanoğlu Ş.F., Demir O., Kolano B., Tuna M. (2026). Nuclear DNA Content Analyses by Flow Cytometry of Saffron (*Crocus sativus* L.) Populations Obtained from Safranbolu, Türkiye. Curr. Issues Mol. Biol..

[26] Bennett M.D., Bhandol P., Leitch I.J. (2000). Nuclear DNA Amounts in Angiosperms and their Modern Uses—807 New Estimates. Ann. Bot..

[27] Bou Dagher-Kharrat M., Abdel-Samad N., Douaihy B., Bourge M., Fridlender A., Siljak-Yakovlev S., Brown S.C. (2013). Nuclear DNA C-values for biodiversity screening: Case of the Lebanese flora. Plant Biosyst.—Int. J. Deal. Asp. Plant Biol..

[28] Bennett M.D., Leitch I.J. (1995). Nuclear DNA amounts in Angiosperms. Ann. Bot..

[29] Savas Tuna T., Yucel G., Ipek Y., Can B., Tuna M. (2022). Flow Cytometric Characterization of White Clover Populations (*Trifolium repens* L.) Growing in Natural Grasslands of Tekirdag, Turkey. J. Balk. Sci. Technol..

[30] Dolezel J., Greilhuber J., Suda J. (2007). Estimation of nuclear DNA content in plants using flow cytometry. Nat. Protoc..

[31] Kolano B., Siwinska D., Gomez Pando L., Szymanowska-Pulka J., Maluszynska J. (2012). Genome size variation in *Chenopodium quinoa* (Chenopodiaceae). Plant Syst. Evol..

[32] Savaş Tuna G.S., Başer İ., Tuna M. (2019). Genome size variation among natural populations of *Brachypodium distachyon* and *B. hybridum* collected from different regions of Turkey. Turk. J. Bot..

[33] Han T.S., Zheng Q.J., Lv J.X., Li S., Du Z.Q., Xing Y.W. (2025). Intraspecific Genome Size Variation in *Rorippa indica* Reveals a Tropical Adaptation by Genomic Enlargement. Physiol. Plant..

[34] Hrabovský M., Kubalová S., Mičieta K., Ščevková J. (2024). Environmental impacts on intraspecific variation in *Ambrosia artemisiifolia* genome size in Slovakia, Central Europe. Environ. Sci. Pollut. Res. Int..

[35] Li M., Wang M., Zhang J., Feng G., Noor M., Guo Z., Guo Y., Guan Y., Yan X. (2023). Cytological Pattern Reveals Genome Downsizing in *Cynodon dactylon* (L.) Pers along the Longitudinal Gradient. Agronomy.

[36] Ling W. (2005). The Relationship between Genome Size and Plant Adaptability to the Environment. MedScien.

[37] Xiao Q.S., Fér T., Guo W., Chen H.F., Li L., Zhao J.L. (2025). Small Genome Size Ensures Adaptive Flexibility for an Alpine Ginger. Genome Biol. Evol..

[38] Meyerson L.A., Cronin J.T., Lučanová M., Lambertini C., Brix H., Packer J.G., Čuda J., Wild J., Pergl J., Pysek P. (2024). Some like it hot: Small genomes may be more prevalent under climate extremes. Biol. Invasions.

[39] Bramel P.J., Volk G. (2019). A global strategy for the conservation and use of apple genetic resources. Glob. Crop Divers. Trust.

[40] Kaçal E., Şimsek Y.I., Seymen T., Özongun Ş., Tuna M. (2023). Nuclear DNA content and ploidy levels of living apple germplasm collection in Türkiye. Turk. J. Bot..

[41] Luo D., Zeng Z., Wu Z., Chen C., Zhao T., Du H., Miao Y., Liu D. (2023). Intraspecific variation in genome size in *Artemisia argyi* determined using flow cytometry and a genome survey. 3 Biotech.

[42] Yurkevich O.Y., Kirov I.V., Bolsheva N.L., Rachinskaya O.A., Grushetskaya Z.E., Zoschuk S.A., Samatadze T.E., Bogdanova M.V., Lemesh V.A., Amosova A.V. (2017). Integration of Physical, Genetic, and Cytogenetic Mapping Data for Cellulose Synthase (*CesA*) Genes in Flax (*Linum usitatissimum* L.). Front. Plant Sci..

[43] Mohapatra P., Ray A., Sriram Sandeep I., Parida R., Mohanty S. (2021). Genetic and biochemical stability of in vitro raised and conventionally propagated Centella asiatica—A valuable medicinal herb. South Afr. J. Bot..

[44] Krahulcova A., Rotreklova O. (2010). Use of flow cytometry in research on apomictic plants. Preslia.

[45] Tomaszewska P., Pellny T.K., Hernández L.M., Mitchell R.A.C., Castiblanco V., de Vega J.J., Schwarzacher T., Heslop-Harrison P. (2021). Flow Cytometry-Based Determination of Ploidy from Dried Leaf Specimens in Genomically Complex Collections of the Tropical Forage Grass *Urochloa* s. l. Genes.

[46] Kavane A., Bishoyi A.K., Geetha K.A. (2021). Assessment of facultative apomixis in *Commiphora wightii* (Burseraceae): A detailed study by controlled pollination followed by histology and flow cytometry. Flora.

[47] Evans G.M., Durrant A., Rees H. (1966). Associated Nuclear Changes in the Induction of Flax Genotrophs. Nature.

[48] Evans G.M. (1968). Nuclear changes in flax. Heredity.

[49] Chen Y., Schneeberger R.G., Cullis C.A. (2005). A site-specific insertion sequence in flax genotrophs induced by environment. New Phytol..

[50] Goldsbrough P.B., Ellis T.H.N., Cullis C.A. (1981). Organization of the 5S RNA genes in flax. Nucleic Acids Res..

[51] Cullis C.A. (2005). Mechanisms and Control of Rapid Genomic Changes in Flax. Ann. Bot..

[52] Dolezel J., Sgorbati S., Lucretti S. (1992). Comparison of three DNA fluorochromes for flow cytometric estimation of nuclear DNA content in plants. Physiol. Plant..

[53] Tiryaki I., Tuna M. (2012). Determination of intraspecific nuclear DNA content variation in common vetch (*Vicia sativa* L.) lines and cultivars based on two distinct internal reference standards. Turk. J. Agric. For..

[54] Realini M.F., Poggio L., Cámara-Hernández J., González G.E. (2016). Intra-specific variation in genome size in maize: Cytological and phenotypic correlates. AoB Plants.

[55] Savaş Tuna G.S., Yücel G., Aşçioğul T.K., Ateş D., Eşiyok D., Tanyolaç M.B., Tuna M. (2020). Molecular cytogenetic characterization of common bean (*Phaseolus vulgaris* L.) accessions. Turk. J. Agric. For..

[56] Knight C.A., Molinari N.A., Petrov D.A. (2005). The large genome constraint hypothesis: Evolution, ecology and phenotype. Ann. Bot..

[57] Li W., Liu L., Wang Y., Fan G., Zhang S., Wang Y., Liao K. (2020). Determination of genome size and chromosome ploidy of selected taxa from *Prunus armeniaca* by flow cytometry. Sci. Hortic..

[58] Kang M., Tao J., Wang J., Ren C., Qi Q., Xiang Q.Y., Huang H. (2014). Adaptive and nonadaptive genome size evolution in Karst endemic flora of China. New Phytol..

[59] Schmuths H., Meister A., Horres R., Bachmann K. (2004). Genome size variation among accessions of *Arabidopsis thaliana*. Ann. Bot..

[60] Simpson K.J., Mian S., Forrestel E.J., Hackel J., Morton J.A., Leitch A.R., Leitch I.J. (2024). Bigger genomes provide environment-dependent growth benefits in grasses. New Phytol..

[61] Potapenko E., Shermeister B., Mandel T., Hübner S. (2026). Intraspecific genome size variation is attributed to adaptive silencing of transposable elements in *Hordeum* species. Mol. Biol. Evol..

[62] Rachinskaya O.A., Lemesh V.A., Muravenko O.V., Yurkevich O.Y., Guzenko E.V., Bol’sheva N.L., Bogdanova M.V., Samatadze T.E., Popov K.V., Malyshev S.V. (2011). Genetic polymorphism of flax *Linum usitatissimum* based on the use of molecular cytogenetic markers. Russ. J. Genet..

[63] Hu T.T., Pattyn P., Bakker E.G., Cao J., Cheng J.F., Clark R.M., Fahlgren N., Fawcett J.A., Grimwood J., Gundlach H. (2011). The *Arabidopsis lyrata* genome sequence and the basis of rapid genome size change. Nat. Genet..

[64] Devos K., Brown J.K., Bennetzen J.L. (2022). Genome size reduction through illegitimate recombination counteracts genome expansion in Arabidopsis. Genome Res..

[65] González L.G., Deyholos M.K. (2012). Identification, characterization and distribution of transposable elements in the flax (*Linum usitatissimum* L.) genome. BMC Genom..

[66] Yi L., Sa R., Mu Y., Zhang J., He R., Song Y., Zhou Y., Chen Y. (2026). Comparative genomics across *Linum* illuminates the evolutionary basis of flax domestication, genome diversification, and seed oil biosynthesis. Front. Genet..

[67] Çatal Ö.F. (2026). Determination of Nuclear DNA Amount of Some Flax Genotypes (*Linum usitatissimum* var. *usitatissimum*) by Flow Cytometry and Investigation of Their Phylogenetic Relationships Using nrITS Markers. Master’s Thesis.

[68] Dolezel J., Bartos J. (2005). Plant DNA flow cytometry and estimation of nuclear genome size. Ann. Bot..

[69] Virtanen P., Gommers R., Oliphant T.E., Haberland M., Reddy T., Cournapeau D., Burovski E., Peterson P., Weckesser W., Bright J. (2020). SciPy 1.0: Fundamental Algorithms for Scientific Computing in Python. Nat. Methods.

[70] Seabold S., Perktold J. Statsmodels: Econometric and statistical modeling with python. Proceedings of the 9th Python in Science Conference.

[71] Hunter J.D. (2007). Matplotlib: A 2D graphics environment. Comput. Sci. Eng..

[72] McKinney W. Data structures for statistical computing in python. Proceedings of the 9th Python in Science Conference.

[73] Shapiro S.S., Wilk M.B. (1965). An analysis of variance test for normality (complete samples). Biometrika.

[74] Kruskal W.H., Wallis W.A. (1952). Use of ranks in one-criterion variance analysis. J. Am. Stat. Assoc..

[75] Ostertagova E., Ostertag O., Kováč J. (2014). Methodology and application of the Kruskal-Wallis test. Appl. Mech. Mater..

[76] Ruxton G.D., Beauchamp G. (2008). Time for some a priori thinking about post hoc testing. Behav. Ecol..

[77] Agbangba C.E., Aide E.S., Honfo H., Kakai R.G. (2024). On the use of post-hoc tests in environmental and biological sciences: A critical review. Heliyon.

[78] Sokal R.R., Michener C.D. (1958). A statistical method for evaluating systematic relationships. Univ. Kans. Sci. Bull..

[79] Odong T.L., Van Heerwaarden J., Jansen J., van Hintum T.J., Van Eeuwijk F.A. (2011). Determination of genetic structure of germplasm collections: Are traditional hierarchical clustering methods appropriate for molecular marker data?. Theor. Appl. Genet..

[80] Hasterok R., Draper J., Jenkins G. (2004). Laying the cytotaxonomic foundations of a new model grass, *Brachypodium distachyon* (L.) Beauv. Chromosome Res..

